# Work experience was associated with the knowledge and practice of catheter-associated urinary tract infection prevention among nurses at University of Gondar Comprehensive Specialized Hospital, northwest Ethiopia, 2021

**DOI:** 10.1186/s12905-023-02176-8

**Published:** 2023-01-30

**Authors:** Alebachew Ferede Zegeye, Chanyalew Worku Kassahun, Yemataw Zewdu Temachu

**Affiliations:** 1 Department of Medical Nursing, School of Nursing, University of Gondar, Gondar, Ethiopia; 2 Department of Emergency and Critical Care, School of Nursing, University of Gondar, Gondar, Ethiopia

**Keywords:** Catheter-associated urinary tract infection, Ethiopia, Knowledge, Nurses, Practice

## Abstract

**Background:**

Catheter-associated urinary tract infection is the source of about 20% of episodes of health-care acquired infections in acute care facilities and it is over 50% in long-term care facilities. In Ethiopia, there is no published scientific research regarding knowledge and practice of nurses on catheter-associated urinary tract infections prevention. Therefore, this study aimed to assess knowledge, practice and associated factors of CAUTI prevention among nurses working at university of Gondar comprehensive specialized hospital, northwest Ethiopia, 2021.

**Methods:**

Institutional based cross sectional study was conducted at University of Gondar Comprehensive Specialized Referral Hospital from April 01 to May 01, 2021 among 423 nurses. Simple random sampling technique was used. Data were collected by using self-administered questionnaire then coded and entered into EPI- Data version 4.6 and then exported to SPSS version 23. Descriptive statistics was computed, and the result was summarized by texts, tables, and charts. All variables with *P*-value < 0.25 in the univariate analysis were included in the multivariable regression analysis. The degree of association was interpreted by using the adjusted odds ratio with 95% confidence intervals.

**Results:**

Good knowledge and practice of nurses towards CAUTI prevention was 37.7% and 51.8% respectively. Good knowledge was associated with sex [AOR = 1.84, 95% CI (1.09, 3.11)], work experience [AOR = 2.36, 95% CI (1.09, 5.10)], working unit [AOR = 6.9, 95% CI (2.11, 22.52)], training [AOR = 2.33, 95% CI (1.17, 4.65)] and had guideline [AOR = 2.78, 95% CI (1.59, 4.88)]. Good practice was associated with sex, work experience, attitude and knowledge towards CAUTI prevention [AOR = 2.19, 95% CI (1.23, 3.88)], [AOR = 5.44, 95% CI (1.66, 17.84)], [AOR = 3.06, 95% CI (1.65, 5.67)], and [AOR = 5.28, 95% CI (2.86, 9.76)] respectively.

**Conclusions:**

Nearly one-third of nurses had good knowledge and more than half of nurses had good practice towards CAUTI prevention. Sex, work experience, work unit, presence of guideline, and training were significantly associated with knowledge. Sex, work experience, good attitude and, knowledge were associated with practice of nurses towards CAUTI prevention. The knowledge and practice towards catheter associated urinary tract infection prevention among nurses should be increased, so that the patients could enjoy and maintain the healthy lifestyle.

## Background

Urinary tract infection (UTI) is one of the most common healthcare acquired infection caused by disease-causing organisms in the ordinarily sterile urine or tissues of the genitourinary tract involving the urethra, bladder, and kidneys [1, 2]. Catheter associated urinary tract infection (CAUTI) is urinary tract infection in a person with an indwelling catheter for more than two days and at least one definite sign of UTI such as fever (> 38^0^C), suprapubic tenderness, costovertebral angle pain, and a urine culture positive for no more than two microorganisms (Centers for Disease Control (CDC), 2015[3]. Catheter associated urinary tract infection is resulted from introduction of indwelling catheter into the bladder and it is one of the most common health care acquired infections encountered in clinical practice which has been performed by unsterile technique, and by not taking adequate measures to maintain cleanliness of the urinary catheter during insertion, maintenance, and removal [4]. *E*. coli (21.4%), Enterococcus (14.9%), *Pseudomonas* aeruginosa (10%), *Klebsiella* pneumonia (7.75%), and Enterobacter (4.15%) are some of the organisms that cause CAUTIs [5]. Female gender, extended catheterization, immune-compromised patients, advanced age, and extended ICU stay were risk factors for developing CAUTIs [6].

Globally urinary tract infection affecting more than 150 million people each year [7] and 70–80% of these urinary tract infections are attributed to catheter associated urinary tract infection [8]. Catheter-associated urinary tract infection is the source of about 20% of episodes of health-care acquired infections in acute care facilities and it is over 50% in long-term care facilities [9]. Catheter associated UTIs are the most common hospital acquired infections, and account for 1 million cases per year In the US [10]. Additionally, the use of indwelling urethral catheters increases the risk of UTI occurrence in health-care settings by 14-fold [11]. Catheter-associated urinary tract infections are responsible for prolonged hospital lengths of stay, bacterial resistance, urosepsis, and even cause death [12]. In the US CAUTI accounted for 32% of all hospital acquired infections, making it the most frequent type of infection experienced in the hospital with approximately 449,000 CAUTI incidences with an estimated cost of 450 million dollar per year [13]. In developing countries including Africa, the prevalence and extent of catheter-associated urinary tract infection remains high due to limited resources and deficiency in health care system. In Uganda, one-third of UTI is accounted for CAUTI [14]. In Ethiopia, catheter associated urinary tract infection (CAUTI) is attributed 60.19 cases per 1000 persons-days [15].

The placement, upkeep, and removal of urinary catheters are all crucial tasks performed by nurses [16]. If nurses follow the guidelines for catheter installation and maintain catheters according to evidence-based practices, catheter-associated urinary tract infections are thought to be the most common and preventable infections [17].

In order to prevent catheter-associated urinary tract infections, nurses should be well-versed in infection control techniques when using urethral catheters. They should also follow recommended practices in this regard [18].

Even though 17–69% of catheter-associated urinary tract infections can be prevented by applying infection prevention methods, the knowledge and practice level of many nurses are not well known regarding catheter insertion, maintenance and removal [19]. According to the study conducted in Pakistan the adequate knowledge and practice level of nurses towards CAUTI prevention was 3.13% and 49% respectively [20]. Another study in Saudi Arabia shows that only 0.73% of nurses have adequate knowledge and 55.7% of nurses have good practice regarding CAUTI prevention [21].

Factors such as advanced level of education and long years of work experience are found to be having a positive impact on the level of nurse’s knowledge and practice towards catheter-associated urinary tract infections prevention [19, 22, 23].

In recent years, certain measures have been taken to control CAUTI. Center for disease control (CDC), Infectious Diseases Society of America (IDSA), world health organization (WHO), and department of health in UK have published their efforts towards CAUTI prevention including reducing inappropriate use of urinary catheters, Perform proper techniques for indwelling catheter insertion, implement proper urinary catheter maintenance procedures, and removing urinary catheters in a timely manner [24–27]. Ethiopia has been taken certain measures to tackle CAUTI through applying recommendations such as limiting use of indwelling urinary catheters and applying practices guidelines for insertion, maintenance, and removal of catheter [28].

Even though many efforts have been made to prevent CAUTI, the burden continues to rise. To the best of our knowledge in Ethiopia there is no study conducted regarding nurse’s knowledge and practice towards CAUTI prevention. Thus the current research is aimed to assess knowledge, practice and its associated factors towards CAUTI prevention among nurses at University of Gondar comprehensive specialized hospital.

## Methods

### Study period and area

This study was conducted from April 01 to May 01, 2021 in University of Gondar comprehensive specialized hospital. The hospital is found in Gondar town which is 727 km from Addis Ababa, the capital of Ethiopia and 180 km from Bahir Dar, the capital of Amhara region. The hospital is used as the referral center for more than 5 million catchment populations. It has more than 1000-bed capacity and provides service to over 200,000 patients annually [29]. It provides both specialty and subspecialty services including pediatrics, surgery, gynecology and obstetrics, internal medicine, psychiatry, and ophthalmology in its in-patient and outpatient services. There are around 495 nurses who are working in the hospital. The nurse’s patient-ratio varied within working units and nurses are performing comprehensive cares in each working unit.

### Study design

Institutional based cross sectional study design was employed in University of Gondar Comprehensive Specialized Hospital.

## Source and study population

### Source population

All nurses working at University of Gondar Comprehensive Specialized Hospital.

### Study population

All nurses working at University of Gondar Comprehensive Specialized Hospital who were available at the time of data collection period.

### Inclusion and exclusion criteria

#### Inclusion criteria

All nurses who were working in university of Gondar Comprehensive Specialized Hospital.

### Sample size determination

Sample size was determined by using single population proportion formula by taking the proportion of knowledge and practice (*p*) 50% (because there was no study conducted in our country regarding this title) with expected margin of error (d) 5% just to maximize the sample size and improve the precision of study, 95% confidence interval and non-response rate 10%.

$${\text{n}} = \, \left( {{\text{Z}}_{{\alpha /{2}}} } \right)^{{2}} {\text{p }}\left( {{1} - {\text{p}}} \right)/{\text{d}}^{{2}}$$

$${\text{n}} = \, \left( {{1}.{96}} \right)^{{2}} . \, 0.{5}\left( {{1} - 0.{5}} \right)/ \, \left( {0.0{5}} \right)^{{2}}$$

$${\text{n}} = { 384}$$

$${\text{Non response rate }} = \, \left( {{384}} \right) \, \left( {{1}0\% } \right) \, = { 38}.{4}$$

$${\text{Total sample }}\left( {\text{n}} \right) \, = { 384} + {38}.{4} = {423}$$

$${\text{Where n}} = {\text{ minimum sample size required to the study}}$$

$${\text{d}} = {\text{ margin of error}}$$

$${\text{p}} = {\text{ prevalence of knowledge and practice}}$$

$${\text{Z}}_{{\alpha /{2}}} = {\text{ value of standard normal distribution}}$$

### Sampling technique and procedure

The total number of sample size was obtained from university of Gondar comprehensive specialized hospital in all inpatient and outpatient services. Study participants were selected by using simple random sampling method and were assigned randomly by lottery method. There are a total 495 of nurses in University of Gondar comprehensive specialized hospital. 70 nurses from medical units, 59 from pediatric units, 83 from surgical units, 27 from ICUs, 13 from dialysis unit, 34 from OR units, 31 from recovery units, 20 from fistula unit, 38 from emergency OPD, and 48 from outpatient department nurses were selected by using proportional allocation. And the total sample size was 423.

### Data collection tool and procedure

A structured English version self-administered questionnaire was used as instrument to collect data which is adopted from different literatures. The data were collected by three trained BSc unemployed nurses for duration of one month and supervised by one midwifery professional [17, 21, 30]. The questionnaire had five sections. The first section dealing with demographic characteristics of respondents, the second deals with work related characteristics, and the third section was about personal factors towards CAUTI prevention.

The fourth section was about 10 questions related to catheter indications, maintenance, care, removal, risk factors for CAUTI, and complications of CAUTI to assess nurses’ knowledge towards CAUTI prevention and each correct answer was scored one and each wrong answer was scored zero. The total score for correct answers from all of the items was computed and classified as follows; good knowledge when study participants respond 71% and above the score of knowledge related Questions and poor knowledge when study participants respond below 71% from knowledge related questions towards CAUTI prevention.

The fifth segment discusses the range of nurses' CAUTI prevention practice. It has 15 questions: five on nursing practices prior to catheter placement, two on nursing practices during catheter insertion, and eight on nursing practices following catheter insertion. Participants in the study were required to select the proper response to each of the questionnaire's questions. Every right response received one point, while every wrong response received zero. The total score for correct answers across all items was calculated, and it was divided into two categories: good practice and poor practice. Good practice is when nurses scored 79.9% and above practice related questions aimed at preventing CAUTIs, and poor practice is when they scored below 79.9% of practice related questions.

The questionnaire was validated by following face validity method. To test the reliability of the tool, Cronbach alpha were calculated after a pre-test by taking 5% of the sample size at Tibebe Ghion specialized hospital and the value was 0.79, 0.83 and 0.89 for knowledge, attitude and practice tools respectively that were in acceptable ranges. The participants involved in the pre-test study were not included in the present study.

## Study variables

### Dependent variables

- Knowledge of nurses towards CAUTI prevention
- Practice of nurses towards CAUTI prevention

## Independent variables

- Socio-demographic variables
  - Age, sex, religion, ethnicity, level of education, marital status, monthly income

- Work related variables
  - Work experience, working unit, training on CAUTI prevention, number of attending training on CAUTI prevention, availability of CAUTI prevention guidelines, usage of CAUTI prevention guidelines

- Personal related variables
  - Attitude of nurses towards CAUTI prevention

### Operational definition

*Knowledge:* is awareness and understanding of nurses regarding CAUTI prevention.

*Good Knowledge:* Nurses who scored 71% and above on knowledge related questions were categorized as having “good knowledge” [31].

*Poor knowledge:* Nurses who scored below 71% on knowledge related questions were categorized as having “poor knowledge” [31].

*Practice:* In this study it refers to the nurses actions regarding CAUTI prevention.

Good Practice: Nurses who scored 79.9% and above on practice related questions were categorized as “good practiced” [17].

*Poor Practice:* Nurses who scored below 79.9% on practice related questions were categorized as “poor practiced [17].

*Favorable attitude:* Refers to those study participants who scored point greater than or equal to the mean score of attitude questions about CAUTI prevention[30].

*Unfavorable attitude*: Refers to those study participants who scored point less than the mean score of attitude questions about CAUTI prevention [30].

### Data processing and analysis

The data were coded, entered, and exported from Epi-data version 4.6 for analysis into the statistical package for social science (SPSS) version 23. Prior to performing regression analysis, the data were cleaned, and then frequencies and cross-tabulation were calculated. Descriptive statistics were calculated, and the results were presented as mean and standard deviation in texts, tables, and charts. The model's fitness was evaluated using the Hosmer–Lemeshow test. Using a univariate logistic regression model, the relationship between each independent variable and the dependent variable was examined. The multivariable regression analysis included every variable in the univariate analysis with a P-value of less than 0.25. The significance level was set at P-value 0.05 in the multivariable logistic regression analysis, and the adjusted odds ratio with 95% confidence intervals was used to determine the degree of association.

### Data quality assurance

Data quality was ensured by providing half day training to data collectors about the research objective, participant selection method, eligible study subjects, data collection tools, and procedures. The data collection tool was adopted from different literatures which was developed in English language and was checked for its consistency in the meaning of words. A pretest was done on five percent [22] from the total sample size at Tibebe Ghion specialized hospital two weeks before the actual data collection period. Based on the result of the pretest, necessary corrections and amendments were taken on the data collection tools (i.e. knowledge related question). Regular supervision was held during the data collection period and the collected data were checked on daily basis for completeness.

## Results

### Socio-demographic characteristics of nurses

A total of 406 participants were included in this study with a response rate of 96%. Among respondents 221 (54.4%) were female. In this study the mean age of the participants was 31.36 years ± 4.03 SD. Most of the participants 306(75.4%) were orthodox Christian and 239(58.8%) were married and 380(93.6%) had more than 5000 birr income and 307(75.6%) nurses had a bachelor’s degree (Table 1).

**Table 1:** Socio-demographic characteristics of nurses to assess knowledge, practice and associated factors towards CAUTI prevention among nurses at UoGCSH, Northwest Ethiopia, 2021 (*N* = 406)

Socio-demographic variables	Frequency(*n*)	Percent (%)
Age		
20–29	143	35.2
30–39	243	59.9
≥ 40	20	4.9
Sex		
Male	185	45.6
Female	221	54.4
Marital status		
Single	162	39.9
Married	244	60.1
Level of education		
Diploma	9	2.2
BSc degree	307	75.6
Masters and above	90	22.2
Monthly income		
< 5000 ETB	26	6.4
5000 – 7000 ETB	201	49.5
7001 – 9000 ETB	156	38.4
> 9000 ETB	23	5.7

*NB. BSc* Bachelor of Science, *ETB* Ethiopian birr

### Work related characteristics of nurses on prevention of CAUTI

More than half of study participants 204(50.2%) had years of experience ranged from 5 to 10 years. Out of 406 nurses who participated in the study, 89(21.9%) nurses were working in major and minor OR, and while 36(9.1%) of nurses were working in Adult emergency OPD. More than half of study participants 223(54.9%) attended training program regarding catheter associated UTI and 26(6.8%) nurses attended training program on catheter associated UTI prevention more than once. More than half of study participants 218(53.7%) had CAUTI prevention guidelines in their working unit (Table 2).

**Table 2:** work related factors to assess knowledge, practice and associated factors towards CAUTI prevention among nurses at UoGCSH, Northwest Ethiopia, 2021 (*N* = 406)

Work related variables	Frequency (*n*)	Percent (%)
Work experience		
< 5 years	116	28.6
5 to 10 years	204	50.2
> 10 years	86	21.2
Attend training on CAUTI prevention		
Yes	223	54.9
No	183	45.1
Number of attending training		
Only once	197	48.5
More than once	26	6.4
Working unit		
Medical units	67	16.5
Surgical units	82	20.2
OR	89	21.9
Pediatric units	58	14.3
Fistula unit	33	8.1
EOPD	37	9.1
Others	40	9.9
Presence of CAUTI prevention guidelines		
Yes	218	53.7
No	188	46.3
Following of CAUTI prevention guidelines		
Yes	204	50.2
No	202	49.8

*NB. Others* *Obstetrics ward *Malnutrition ward *Oncology unit *Chronic OPD

*OR *Operating room, *EOPD* Emergency out patient department

### Attitude of nurses towards prevention of CAUTI

From a total of 406 nurses 53.4% had good attitude and 46.6% had poor attitude towards CAUTI prevention (Table 3).

**Table 3:** Frequency distribution of participants response of Attitude towards CAUTI prevention among nurses at UoGCSH, Northwest Ethiopia, 2021 (*N* = 406)

Questions	Strongly agree		Agree		Neutral		Disagree		Strongly disagree	
	*N*	%	*N*	%	*N*	%	*N*	%	*N*	%
It is difficult to keep track of catheters placed	37	9.1	151	37.2	21	5.2	156	38.4	41	10.1
Development of CAUTI cannot be avoided	43	10.6	121	29.8	38	9.4	139	34.2	65	16.0
It is unrealistic to clean hands after every contact with patient	69	17.0	122	30.0	28	6.9	121	29.8	66	16.3
Insertion of catheters should be for nursing convenience	57	14.0	157	38.7	27	6.7	100	24.6	65	16.0
I do not like taking care of patients in need of catheters	34	8.4	119	29.3	22	5.4	140	34.5	91	22.4
I do not have time to follow the guidelines	100	24.6	75	18.5	15	3.7	168	41.4	48	11.8

### Nurses’ Knowledge regarding to prevention of CAUTI

Participants were asked 10 questions to assess their knowledge on CAUTI prevention, and they were categorized into two groups based on their score. More than half of 253(62.3%) of the study participants had poor level of knowledge, while nearly one-third of nurses 153(37.7%) had good level of knowledge towards prevention of CAUTIs. The mean knowledge score of the study participant was 5.64 (SD ± 2.49) (Table 4).

**Table 4:** Frequency distribution of participants response of knowledge towards CAUTI prevention among nurses at UoGCSH, Northwest Ethiopia, 2021 (*N* = 406)

Questions	Yes		No	
	Freq	%	Freq	%
Among the following what is an inappropriate indication for indwelling urinary catheterization	210	51.7	196	48.3
Which is an appropriate indication of urinary catheterization among the following	198	48.8	208	51.2
Proper technique used for indwelling urinary catheter Insertion	296	72.9	110	27.1
Based on CDC 2009 Guidelines for prevention of CAUTI, it is advised to remove the catheter as soon as possible post operatively, preferably with in	221	54.4	185	45.6
If catheter is obstructed during you are assess patient, what are you going to do	254	62.6	152	37.4
One of the following is not a nursing action to prevent infections from urinary catheter	209	51.5	197	48.5
Which one among the following is a risk factor for CAUTI	238	58.6	168	41.4
Which patient is at high risk of mortality or developing CAUTI	214	52.7	192	47.3
Which action is perform before catheter insertion from the following except	203	50.0	203	50.0
All the following are complications of CAUTI except	247	60.8	159	39.2

### Nurses’ practice regarding to prevention of CAUTI

By using 15 practice based questions, Nearly one-third 210(51.8%) of the respondents had good practice; whereas the remaining 196(48.2%) respondents had poor practice of CAUTI prevention the mean practice score of the respondents was found to be 10.22 (SD ± 3.27) (Table 5).

**Table 5:** Frequency distribution of participants response of practice towards CAUTI prevention among nurses at UoGCSH, Northwest Ethiopia, 2021 (*N* = 406)

Questions	Yes		No	
	Freq	%	Freq	%
Do you wash your hands before you insert the catheter	380	93.6	26	6.6
What type of gloves do you use when you insert the catheter	297	73.2	109	26.8
Do you use sterile lubricant	376	92.6	30	7.4
What type of solution do you use to clean the urethra before you insert the catheter	251	61.8	155	38.2
How do you open and handle the indwelling catheter from its packaging	223	54.9	183	45.1
What type of solution do you inject in the needleless port to inflate the balloon	333	82.0	73	18.0
Do you secure indwelling catheter after insertion to prevent movement of urinary catheter	301	74.1	105	25.9
Do you keep the catheter and collecting tube free from kinking	301	74.1	105	25.9
How do you keep the urine collecting bag	215	53	191	47.0
Do you empty the collecting bag regularly and use a separate, clean urine collecting jug for each patient	295	72.7	111	27.3
How do you maintain the system	198	48.8	208	51.2
Do you keep the urine collecting bag out off the floor	308	75.9	98	24.1
Do you avoid contact of the drainage spigot with the collecting container	258	63.5	148	36.5
Do you cleaning the perianal area during daily bathing or showering	220	54.2	186	45.8
What do you use during any manipulation of the catheter or collecting system	195	48.0	211	52.0

### Factors associated with the knowledge of nurses towards CAUTI prevention

Sex, work experience, working unit, presence of guideline, and training on prevention of catheter associated urinary tract infection were found to have significant association with nurses’ knowledge regarding to prevention of CAUTI.

For males the odds of good knowledge towards catheter associated urinary tract infection prevention was 1.84 times that of females [AOR = 1.84, 95% CI (1.09, 3.11)]. The odds of good knowledge towards catheter associated UTI prevention among nurses with 5 to 10 years work experience was 2.36 times that of nurses with < 5 years of work experience [AOR = 2.36, 95% CI (1.09, 5.10)]. The odds of good knowledge towards catheter associated UTI prevention among nurses working in emergency OPD was 6.9 times that of working in medical units [AOR 6.9, 95% CI (2.11, 22.52)]. The odds of good knowledge towards catheter associated UTI prevention among nurses who took training related to catheter associated UTI was 2.33 times that of nurses who had not taken [AOR = 2.33, 95% CI (1.17, 4.65)]. For nurses who had catheter associated UTI prevention guidelines the odds of good knowledge towards CAUTI prevention was 2.78 times that of nurses who had not [AOR = 2.78; 95%CI (1.59, 4.88)] (Table 6).

**Table 6:** Factors Associated with the Knowledge of Nurses towards CAUTI prevention at UoGCSH, Northwest Ethiopia, 2021 (N = 406)

Variables	Knowledge		COR (95% CI)	AOR (95% CI)
	Good	Poor		
	*N*	*N*		
Age (in years)				
20 -29	107	36	1	1
30 -39	76	167	6.53(4.10, 10.40)	2.04(0.99, 4.17)
> 40	8	72	4.46(1.69, 11.77)	0.52(0.13, 2.05)
Sex				
Male	115	70	1.99(1.335, 2.96)	**1.84 (1.09, 3.11)**
Female	100	121	1	1
Monthly income				
< 5000 ETB	18	8	1	1
5001–7000 ETB	130	71	1.23(0.51, 2.97)	0.49(0.18, 1.32)
7001–9000 ETB	40	116	6.53(2.63, 16.16)	1.12(0.38, 3.32)
> 9000 ETB	3	20	15.00(3.44, 65.36)	4.99(0.83, 29.98)
Work experience				
< 5 years	93	23	1	1
5 to 10 years	74	130	7.10(4.15, 12.17)	**2.36(1.09, 5.10)**
> 10 years	24	62	10.45(5.42, 20.13)	1.25(0.44, 3.57)
Attend training on CAUTI prevention				
Yes	168	55	8.84(5.64, 13.87)	**2.33 (1.17, 4.65)**
No	47	136	1	1
Working unit				
Medical units	35	32	1	1
Surgical units	53	29	0.60 (0.31, 1.16)	0.49(0.21, 1.14)
OR	39	50	1.40 (0.74, 2.65)	1.51(0.67, 3.40)
Pediatric units	28	30	1.17 (0.58, 2.37)	1.13(0.45, 2.83)
Fistula unit	6	27	4.92 (1.80, 13.46)	2.73(0.81, 9.20)
EOPD	7	30	4.69(1.809, 12.15)	**6.90(2.11, 22.52)**
Others	23	17	0.81(0.37, 1.78)	0.76(0.29, 2.00)
Presence of CAUTI prevention guidelines				
Yes	156(38.4)	62(15.3)	5.50(3.59, 8.42)	**2.78 (1.59, 4.88)**
No	59(14.5)	129(31.8)	1	1

Bold numbers indicate significantly associated variables with nurses’ knowledge

‘*COR*’ Crude odds ratio, ‘AOR’ Adjusted odds ratio

Hosmer and Lemeshow test = 0.194

### Factors associated with the practice of nurses towards CAUTI prevention

Sex, work experience, good attitude and good knowledge were found to have significant association with practice of nurses’ towards prevention of catheter associated UTI, while presence of guidelines and working unit were not significantly associated at *p*-value of < 0.05.

For males the odds of good practice towards catheter associated UTI prevention was 2.19 times that of females [AOR = 2.19, 95% CI (1.23, 3.88)]. The odds of good practice towards catheter associated UTI prevention among nurses with > 10 years work experience was 5.44 times that of nurses with < 5 years of work experience [AOR = 5.44, 95% CI (1.66, 17.84)]. Moreover the odds of good practice towards catheter associated UTI prevention among nurses with 5 to 10 years work experience was 3.59 times that of nurses with < 5 years of work experience [AOR = 3.59, 95% CI (1.46, 8.87)]. The odds of good practice towards catheter associated UTI prevention among nurses having favorable attitude was 3.06 times that of nurses having unfavorable attitude [AOR = 3.06, 95% CI (1.65, 5.67)]. The odds of good practice towards catheter associated UTI prevention among nurses having good knowledge was 5.28 times that of nurses having poor knowledge [AOR = 5.28, 95% CI (2.86, 9.76)] (Table 7).

**Table 7:** Factors Associated with the practice of Nurses towards CAUTI prevention at UoGCSH, Northwest Ethiopia, 2021 (*N* = 406)

Variables	Practice		COR (95% CI)	AOR (95% CI)
	Good	Poor		
	*N* (%)	*N* (%)		
Age (in years)				
20 -29	106	37	1	1
30 -39	92	151	4.70(2.98, 7.41)	0.68(0.29, 1.58)
> 40	5	15	8.60(2.92, 25.29)	0.92(0.21, 4.08)
Sex				
Male	115	70	2.48(1.66, 3.71)	**2.19(1.23, 3.88)**
Female	88	133	1	1
Monthly income				
< 5000 ETB	13	13	1	1
5000–7000 ETB	148	53	0.36(0.16, 0.82)	0.24(0.08, 1.74)
7001–9000 ETB	37	119	3.22(1.37, 7.55)	1.04(0.31, 3.50)
> 9000 ETB	5	18	3.60(1.03, 12.62)	0.51(0.09, 2.84)
Work experience				
< 5 years	98	18	1	1
5 to 10 years	86	118	7.47(4.21, 13.27)	**3.59 (1.46, 8.87)**
> 10 years	19	67	19.20(9.39, 39.27)	**5.44 (1.66, 17.84)**
Attend training on CAUTI prevention				
Yes	154	69	6.10(3.96, 9.41)	0.76(0.35, 1.66)
No	49	134	1	1
Working unit				
Medical units	37	30	1	1
Surgical units	51	31	0.75 (0.40, 1.45)	0.65(0.25, 1.70)
OR	34	55	1.99 (1.05, 3.80)	1.26(0.51, 3.14)
Pediatric units	34	24	0.87 (0.43, 1.77)	0..60(0.22, 1.68)
Fistula unit	11	22	2.47 (1.03, 5.88)	0.89(0.28, 2.74)
EOPD	10	27	3.33 (1.39, 7.96)	2.43(0.73, 8.03)
Others	26	14	0.66 (0.30, 1.49)	0.43(0.15, 1.27)
Presence of CAUTI prevention guidelines				
Yes	142	76	3.89(2.57, 5.88)	1.69(0.88, 3.25)
No	61	127	1	1
Attitude				
Poor	139	50	1	1
Good	64	153	6.65(4.30, 10.27)	**3.06(1.65, 5.67)**
Knowledge				
Poor	151	40	1	1
Good	52	163	11.83(7.41, 18.89)	**5.28(2.86, 9.76)**

Bold numbers indicate significantly associated variables with nurses’ Practice Hosmer and Lemeshow test = 0.461

## Discussion

Catheter associated urinary tract infection (CAUTI) is one of major global health concern, which places a huge burden on the health care system. Nurses are expected to play a significant role in the prevention of CAUTIs [32].

The result of this study showed that 37.7% with 95%CI (34.1, 39.2) of study participants had good knowledge about catheter associated urinary tract infection prevention. This finding is in line with study conducted in Egypt (37.3%) [21], India(16.7%) [5], and Rwanda(36.5%) [17]. Even though, there is difference in socioeconomic status and level of health sector development, the possible reason of similarity between the current study and study in Egypt, India, and Rwanda might be using a similar study population (staff nurse), study unit and study design.

The result of this study also lower than the study conducted in Sri Lanka (63.9%) [33], in India (56.7%) [34]. and in Rwanda (64.52%) [17]**.** The possible reason for this difference might be due to difference in study unit and study settings. On this study nurses working in inpatient and outpatient services were included in only one hospital; whereas in study done in Rwanda the study subject was nurses working only in ICUs at two different referral hospital.

However, the result of this study was higher than the study conducted in India 16.7% [5], and in Saudi Arabia (37.23%) [21]. The possible justification might be due to differences in study design, differences in study setting, and the differences in result rating; for example, in Saudi Arabia study, the knowledge was rated as Good, moderate, and poor. As well, the result rating in India’s study is quite different; it was rate as high, moderate and low. This may differ from the figurative interpretation of this study as good and poor knowledge.

Males were 1.84 times more likely than females to have good knowledge about preventing catheter-associated urinary tract infections. [AOR = 1.84, 95% CI (1.09, 3.11)]. This result was in disagreement with the Turkish study [35]. In the Ethiopian context, males may be exposed to more sociocultural and social activities than females, which may be the possible explanation. Additionally, compared to men, women engage in remarkable activities like cooking, cleaning, and preparing food for daily consumption. Consequently, they might not have as much time to read and expand their knowledge [36].

Compared to nurses with less than five years of work experience, nurses with five to ten years of experience had a 2.36 times higher chance of having good knowledge in preventing catheter-associated UTIs. [AOR = 2.36, 95% CI (1.09, 5.10)]. Similar finding was reported in study conducted in India; where years of experience were significantly associated with knowledge [37]. Another study has conducted in Rwanda revealed that, the greater the working experience the higher the knowledge gained [17]. The reason might be that nurses with greater job experience have more opportunities to collaborate with other professions so they can benefit from their colleagues' experiences. Additionally, because the participants have been in their professions for a long time, they have had a variety of exposures that have improved their knowledge of CAUTI prevention in relation to daily activities.

The odds of good knowledge towards catheter associated UTI prevention among nurses who took training related to catheter associated UTI was 2.33 times that of nurses who had not taken [AOR = 2.33, 95% CI (1.17, 4.65)]. This finding was consistent with the study conducted in India [37], Egypt [38]. The possible explanation might be due to that training can enhance an individual’s body of knowledge regarding CAUTI prevention.

The odds of good knowledge towards catheter associated UTI prevention among nurses working in emergency OPD was 6.9 times that of working in medical units [AOR 6.9, 95% CI (2.11, 22.52)]. This finding was consistent with the study conducted in Saudi Arabia [21]. The possible explanation might be because of the prevalence of violence and none violence causality emergency cases occurred more in emergency units than the medical units. Therefore, nurses who are working in emergency ward face patients who need immediate intervention and treatments. They may lead them to read and update themselves.

For nurses who had catheter associated UTI prevention guidelines the odds of good knowledge towards CAUTI prevention was 2.78 times that of nurses who had not [AOR = 2.78; 95%CI (1.59, 4.88)]. This finding was supported by a study conducted in KP Pakistan [39]. This can be justified by the fact that the presence of guideline will advocate the art and science of CAUTI prevention. Availability of CAUTI prevention guideline is likely improving nurses’ knowledge and providing continuity of care.

More than half 210(51.8%) 95% CI (48.6, 55.7) of study participants had good practice towards prevention of catheter associated urinary tract infection. This result was lower than the studies conducted in India (86.5%)[34], Sri Lanka (79.5%)[33], Saudi Arabia (55.7%), [21], and Rwanda (79.9%) [17]**.** The possible justification might be due to difference in socioeconomic status, level of health sector development, study setting, study design, and difference in tool used to rating the outcome.

On the other hand, this study finding was higher than the study conducted in Pakistan (49%) [23], and Egypt (16.1%) [21]. The reason behind this difference might be difference in study setting and the difference in instrument (tool) used to assess nurses’ practice towards prevention of catheter associated urinary tract infection.

In a multivariable logistic regression analysis, variables like sex, work experience, having good attitude and, good knowledge were found to have significantly associated with the practice of nurses towards catheter associated UTI prevention.

For males the odds of good practice towards catheter associated UTI prevention was 2.19 times that of females [AOR = 2.19, 95% CI (1.23, 3.88)]. This finding was inconsistent with the study conducted in Rwanda [17]. The possible explanation might be in Ethiopia, most of the home-based activities such as food preparation and food serving, child feeding, cloth hygiene, and home-based sanitation are left for women. Therefore, women may not get time to practice CAUTI prevention because of being busy taking care of the family members compared with men.

The odds of good practice towards catheter associated UTI prevention among nurses with > 10 years work experience was 5.44 times that of nurses with < 5 years of work experience [AOR = 5.44, 95% CI (1.66, 17.84)], Moreover the odds of good practice towards catheter associated UTI prevention among nurses with 5 to 10 years work experience was 3.59 times that of nurses with < 5 years of work experience [AOR = 3.59, 95% CI (1.46, 8.87)]. Similar finding was reported in studies conducted in India [40], Pakistan [20]. The finding might be justified as nurses’ experience increased; they become familiar with the subject matter thereby acquire good practice to carry out the procedure with safety and precision.

The odds of good practice towards catheter associated UTI prevention among nurses having good knowledge was 5.28 times that of nurses having poor knowledge [AOR = 5.28, 95% CI (2.86, 9.76)]. This finding was consistent with the study conducted in India [41], Iran [42] and Saudi Arabia [21]. The possible explanation could be because of the more knowledgeable to a particular subject matter the more skillful to a particular activity.

The odds of good practice towards catheter associated UTI prevention among nurses having favorable attitude was 3.06 times that of nurses having unfavorable attitude [AOR = 3.06, 95% CI (1.65, 5.67)]. This finding was in line with the study conducted in Tamil Nadu, India [43]. The possible explanation might be individuals with good attitude towards CAUTI prevention have good practice level towards CAUTI prevention.

### Limitation of the study

Despite, the high response rate in this study, social desirability bias and recall bias are potential limitations of these self-reported results.

I.e. study participants might not give true and genuine responses on the self-administered questionnaire, preferring to provide more socially acceptable responses than their actual day to day practice.

## Conclusion

This study revealed that nearly one-third of nurses who working in university of Gondar comprehensive specialized hospital had good knowledge regarding CAUTI prevention. Factors associated with good knowledge towards CAUTI prevention were sex, length of work experience, working unit, presence of guideline, and having training.

More than half of study participants had good practice towards CAUTI prevention. Factors associated with good practice towards CAUTI prevention were sex, work experience, having good attitude and, good knowledge were found to have significantly associated with the practice of nurses towards catheter associated UTI prevention. The knowledge and practice towards catheter associated urinary tract infection prevention among nurses should be increased, so that the patients could enjoy and maintain the healthy lifestyle.

Further studies to exploit factors associated with knowledge and practice of nurses towards CAUTI prevention should be undertaken.

## Data Availability

Data will be available upon request from the corresponding author.

## Abbreviations

CAUTI: Catheter associated urinary tract infection
CDC: Center for disease control
DC: Data collector
ERC: Ethical review committee
HAI: Health care acquired infection
IDSA: Infectious diseases society of America
IUC: Indwelling urinary catheter
KM: Kilometer
KP: Knowledge, practice
NHSN: National healthcare safety network
PI: Principal investigator
SPSS: Statistical package for social science
UoGCSH: University of Gondar comprehensive specialized hospital
UTI: Urinary tract infection
WHO: World health organization
